# Exosomal lncRNA LINC01711 facilitates metastasis of esophageal squamous cell carcinoma via the miR-326/FSCN1 axis

**DOI:** 10.18632/aging.203389

**Published:** 2021-08-09

**Authors:** Mei-Ling Xu, Tian-Cheng Liu, Feng-Xiang Dong, Ling-Xin Meng, Ai-Xia Ling, Shan Liu

**Affiliations:** 1 Department of Oncology, Rizhao People's Hospital, Rizhao, Shandong Province, China; 2 First Department of Oncology, People's Hospital of Juxian, Rizhao, Shandong Province, China; 3 Department of Physical-Chemistry, College of Pharmaceutical Sciences, Jining Medical College, Jinan, Shandong Province, China

**Keywords:** esophageal squamous cell carcinoma, Linc01711, exosome, metastasis, miR-326

## Abstract

Esophageal cancer is a malignant tumor with a five-year survival rate of less than 20%. Early diagnosis and exploration of esophageal cancer pathogenesis are of great significance for the treatment and prognosis of esophageal cancer. Long non-coding RNA (lncRNA) plays a vital role in the occurrence and development of different types of tumors. However, the role of exosome LncRNA in esophageal squamous cell carcinoma (ESCC) is rarely reported. In this study, we detected high expression of lncRNA LINC01711 in ESCC tissues and was associated with poor prognosis. Silencing LINC01711 can inhibit the proliferation, migration, invasion, and growth of ESCC cell lines, and induce apoptosis. Linc01711 was identified as a competitive endogenous RNA that suppressed miR-326, and up-regulated the expression of fascin actin-bundling protein 1 (FSCN1). Besides, *in vivo* experiments showed that the administration of exosome-derived LINC01711 (LINC01711-Exo) promoted the growth of tumors in nude mice. In general, exosomal LINC01711 promoted the proliferation, migration, and invasion of esophageal cancer cells by up-regulating FSCN1 and down-regulating miR-326, thus improved the occurrence and development of ESCC.

## INTRODUCTION

Esophageal cancer ranks the eighth in cancer-related global morbidity and the sixth in mortality [1]. Esophageal squamous cell carcinoma (ESCC) accounts for more than 90% of pathological types in Asian and Western countries [2]. Because the early clinical symptoms are not visible and the lesion anatomical site is unique, most of the patients are at an advanced stage after diagnosis [3]. Many endogenous and exogenous factors could induce DNA damage, such as endogenous and exogenous alkylating agents, and reactive halogen species, and give rise to mutations which can cause progression to cancer [4, 5]. At present, the exact etiology and pathogenesis of esophageal cancer are still unclear. The 5-year overall survival rate of ESCC is less than 20% [2]. Therefore, early diagnosis and exploration of the mechanisms of pathogenesis of ESCC are of great significance.

The exosomes are membrane microbubbles produced by the fusion of intracellular vesicles and plasma membrane, which are about the size of 30-100 nm. There are many exosomes in body fluids such as intercellular space, plasma, urine, and cerebrospinal fluid [6]. Exosomes from different sources contain different components but generally contain proteins, lipids, nucleic acids, and other substances [7]. Exosomes have a unique lipid bilayer membrane structure that can protect these critical vesicular contents, prevent them from being degraded, and target the contents to specific cells or tissues. Exosomes derived from tumor cells can regulate the proliferation and apoptosis of tumor cells and affect tumor invasion and metastasis. These characteristics indicate that exosome detection has excellent potential as a non-invasive fluid biopsy index for cancer [8].

LncRNAs are RNA molecules with a length of more than 200 bp. According to the principle of complementary base pairing, they can specifically bind to nucleic acids [9]. Studies have shown that lncRNA containing exosomes can stably exist in serum, plasma, and other body fluids, and are not affected by endogenous RNases. The concentration of lncRNA in exosomes is up to 4 fold higher than the intracellular. LncRNA is targeted to the receptor cells through exosome, participating in physiological processes such as cell growth and proliferation. The exosome-mediated lncRNA communication plays vital roles in the transduction of biological information between cells [10, 11].

Previous studies reported that there was abnormal expression of a variety of lncRNAs in the primary and metastatic foci of tumors, which may affect the occurrence, development, invasion, and metastasis of tumors [12]. In 2013, Huang et al. [13] confirmed the existence of lncRNA in plasma exosomes for the first time. They detected extracellular RNA in plasma through RNA in-depth sequencing, and found that in exosomes there were not only large number of microRNAs but also lncRNAs, accounting for about 3.36% of total non-coding RNAs.

LncRNA LINC01711 has been identified as an independent prognosis factor of lower-grade gliomas [14]. In this study, we detected lncRNA LINC01711, which is differentially expressed in plasma exosomes derived from ESCC tissue. The aim is to explore whether LINC01711 affects the treatment and prognosis of esophageal cancer, and to clarify its mechanism.

## MATERIALS AND METHODS

### Tissue specimen

The tumor tissues and paired normal adjacent tissues from 137 patients were collected in our hospital, which were used for follow-up experimental detection. The Ethics Review Committee of Rizhao People's Hospital approved the experiment, and all the patients signed informed consent.

### Animals

Animal experiments were permitted by the Animal Protection and Ethics Committee of Rizhao People's Hospital. The research was carried out based on the proposals in the Guidelines for the Care and Use of Laboratory Animals of the National Institutes of Health. BALB/c nude mice were purchased from Weitong Lihua Experimental Animal Technology Co., Ltd., Beijing, China. For the experiment of Xenograft, TE-1 cells (5 × 10^6^) were suspended in 200 μL normal saline and injected subcutaneously. Tumor volumes were calculated by using the formula: Volume (mm^3^) = (L x W x H) / 2, where L was the tumor length, W was the tumor width, H was the tumor height.

### Cell culture

ESC cell lines were purchased from CHI Scientific, Inc., (Jiangsu, China). The cells were cultured in complete medium, including 89% 1640 and 10% FBS, and both were purchased from Biological Industries (Beit-Haemek, Israel). Cells were maintained in a humidified incubator at 37° C with 5% of CO_2_.

### Colony formation

The TE-1 cell suspension was inoculated into a 10-cm dish with 300 cells per dish. After the cells were evenly distributed, they were cultured in a cell incubator for 14 ~ 21 days. When a clone was visible to the naked eye, the medium was abandoned, and 4% paraformaldehyde was added to fix the cells for 15 min. Then the fixed cells were stained with Giemsa for 15 min and rinsed with running water. The number of colonies in each dish was counted with naked eye.

### qRT-PCR

RNA extraction was performed using Trizol reagent. The single-stranded cDNAs were synthesized from RNA. The mRNA and lncRNA levels were quantified by RT-PCR with SYBR Green I (Thermo Fisher Scientific, Inc.). Primers for qPCR were as following. LINC01711 forward 5’-AGGTCAGGCCATACCCA-3’, reverse 5’-CCAGCCATCAGGTTCTGT-3’; U6 forward 5’-AACGAGACGACGACAGAC-3’, reverse 5’-GCAAATTCGTGAAGCGTTCCATA-3’; miR-326, forward 5’-CATCTGTCTGTTGGGCTGGA-3’, reverse 5’-AGGAAGGGCCCAGAGGCG-3’; FSCN1 forward 5’-TGTTTTCGCGAGCAGGTG-3, reverse 5-AGCCTGATTAATTAAGCTATG-3’; GAPDH forward 5’-ACCACAGTCCATGCCATCAC-3’, reverse 5’-TCCACCACCCTGTTGCTGTA-3’.

### RNA immunoprecipitation (RIP) assay

The binding of LINC01711 to Ago2 mRNA was detected by the RIP kit (Millipore, Bedford, MA, USA). All steps were carried out in accordance with the kit instructions. The primary antibody of Ago2 was from Abcam (Cambridge, MA, USA). IgG was used as the control.

### Transfection

Stable TE-1 cell line for overexpression or knockdown of LINC01711 was established by lentivirus transduction (GenePharma, Shanghai, China) and was selected using puromycin. miR-326 mimics, inhibitor and the control sequences were from Ribo (Ribo Bio, Guangzhou, China). miR-mimics and inhibitors were transiently transfected in TE-1 cells with lipofectamine 2000 (Invitrogen, Waltham, MA, USA).

### Western blot

After RIPA lysis, total proteins were extracted and measured with BCA method. Protein was separated by SDS-PAGE and then transferred onto a nitrocellulose (NC) membrane and blocked for 1 h at room temperature with 5% skim milk. Then primary antibodies were added, and placed overnight at 4° C. The NC membrane was washed three times with TBST solution, then added with goat anti-rabbit secondary antibody (horseradish peroxidase (HRP) conjugate), and left to stand for 1 h at room temperature. Finally, the membrane was washed with TBST again and visualized with enhanced chemiluminescence (ECL) reagent. The relative expression level of the protein was presented as the gray value of test band/gray value of the β-actin band. The primary antibody of CD63 (1:500), CD9 (1:500), CD81 (1:300) and FSCN1 (1:500) were purchased from ProteinTech (Wuhan, China).

### TEM observation

Exosome samples were prepared as described earlier [15]. The samples were observed under a transmission electron microscope (JEM-2100F, Tokyo, Japan).

### Fluorescence *in situ* hybridization (FISH) assay

The probes of LINC01711, 18s rRNA and U6 RNA were synthesized by Ribo Bio (Guangzhou, China). FISH *in situ* hybridization kit (Ribo Bio, Guangzhou, China) was used for detection. Nucleus was stained with DAPI. The pictures were observed and photographed with Olympus fluorescence microscope.

### Cell apoptosis assay

Cells were stained according to the instructions of the apoptosis detection kit. Apoptosis was detected by a post-flow cytometer. The results consisted of four quadrants, including healthy cells [An(-)/PI(-), at the lower left quadrant], early apoptotic cells [An(+)/PI(-), at the lower right], late apoptosis and necrosis cells [An(+)/PI(-), at the upper right], and then the dead cells [An(-)/PI(+), at the top left], and the apoptosis rate was calculated as follows: the ratio of early apoptosis plus the proportion of late apoptotic cells to the total cells.

### Dual luciferase reporter assay

The intact 3’UTR (wild-type and mutant type) of LINC01711 or FSCN1 was synthesized and inserted into the down-stream of the luciferase gene of the dual-luciferase reporter plasmid pMIR vector, respectively. All the constructs were verified by sanger sequencing. The wild-type or mutant luciferase reporter plasmid were co-transfected with miR-326 mimics or miR-NC (GenePharma, Shanghai, China) into TE-1 cells using Lipofectamine2000. The sequences were as follows: miR-326 mimics, 5’-CCUCUGGGCCCUUCCUCCAG-3’; miR-NC mimics, 5’-UCGCUUGGUGCAGGUCGGGAA-3’. At 48 h post-transfection, cells were assayed for luciferase activity using the Dual Luciferase Assay System (Promega, Madison, WI, USA) according to the manufacturer’s instructions.

### EdU assay

EdU Cell Proliferation Kit (RiboBio, Guangzhou, China) was used to test cell proliferation. TE-1 cells were seeded in 24-well plates for transfection. Cells were maintained with 200 μL 50 μM EdU for 2 h. Apollo Dye Solution (red) were used to stain actively proliferating cells, nucleic acids were stained with DAPI (blue) according to the protocols and then photographed.

### Wound-healing assay

TE-1 cells were plated in 6-well plates until the cells formed a confluent monolayer, then scratched using a 100 μL pipette tip. The scratch wounds were captured using microscopy immediately after scratching and 24 h later. The wound area was analyzed by ImageJ.

### Matrigel transwell assay

24-well matrigel transwell (Corning, NY, USA) were used to investigated cell invasion. 2 × 10^5^ TE-1 cells were seeded in the cell culture inserts precoated with 1 μg/μL Matrigel (BD Biosciences, NJ, USA). Medium include 20% FBS was used to stimulate invasion in the bottom of wells. After 48 h, the invasion cells were stained with 0.1% crystal violet solution.

### Statistical analysis

Data were shown as Mean ± SD. Student's *t*-test was used to compare two groups and one-way ANOVA for multiple groups comparison. *p*<0.05 was considered statistically significant.

### Data availability statement

The original contributions presented in the study are included in the article/supplementary material, further inquiries can be directed to the corresponding authors.

## RESULTS

### Up-regulation of LINC01711 was detected in ESCC

**Figure 1:**
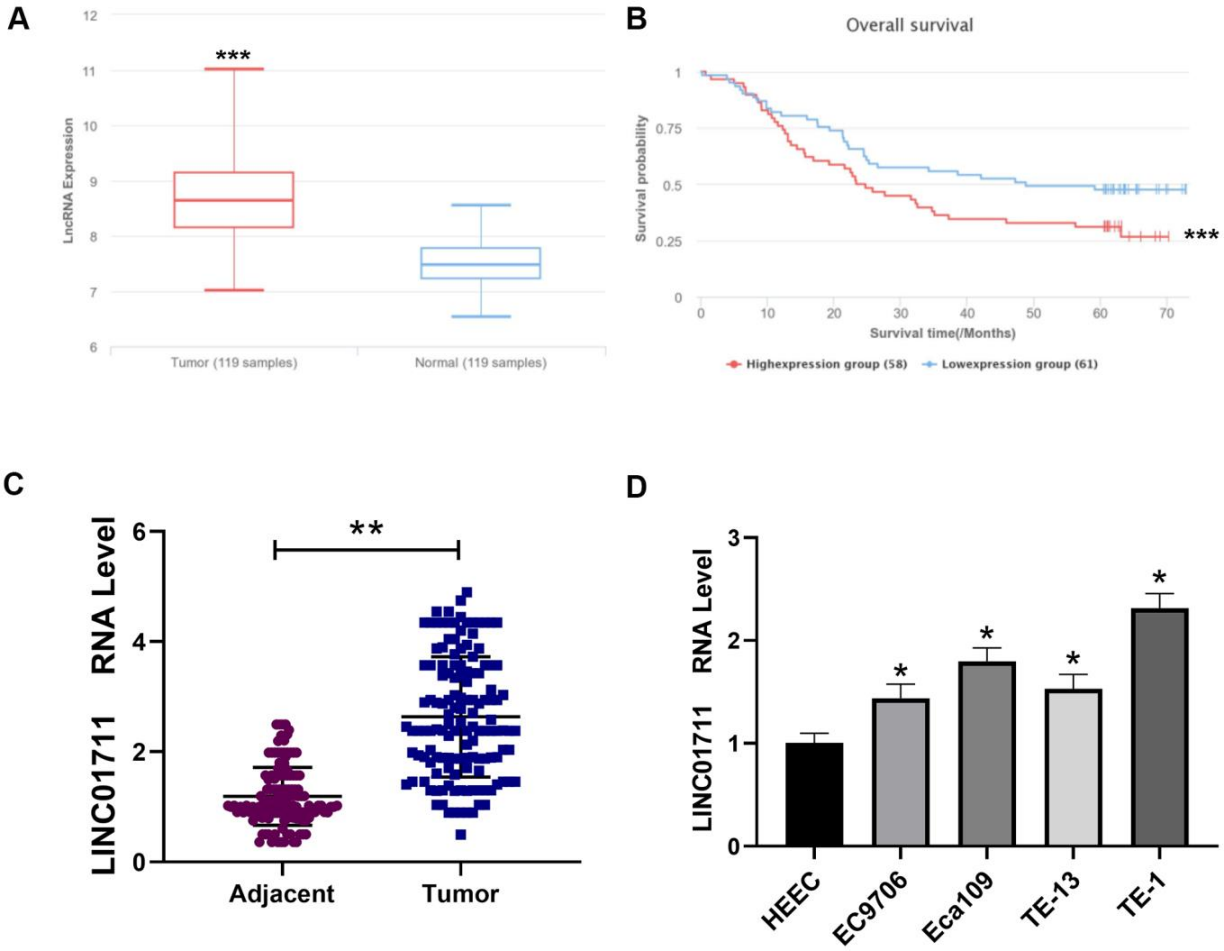
**Linc01711 was highly expressed in ESCC.** (**A**) The expression of LINC01711 in ESCC and adjacent normal tissues was predicted by LnCAR database. (**B**) The expression of LINC01711 was related to the survival of patients predicted by LnCAR database. (**C**) The LINC01711 levels in ESCC and adjacent normal tissues were detected by RT-qPCR (n=137). (**D**) RT-qPCR was used to test the LINC01711 level in human normal esophageal epithelial cell line HEEC and five esophageal squamous carcinoma cell lines. n=4. *p* < 0.05; ** *p* < 0.01; *** *p* < 0.001.

To see the expression profile of LINC01711 in ESCC, we first searched the public microarray data in LnCAR database (https://renlab.org) [16]. We found that LINC01711 was highly expressed in ESCC (Figure 1A), and the level of LINC01711 was associated with a low survival rate (Figure 1B). Next, we checked the expression level of LINC01711 in paired ESCC tissue samples and normal adjacent tissue samples from 137 ESCC patients by RT-qPCR. We found that the level of LINC01711 in esophageal squamous cell carcinoma was higher than that in adjacent healthy tissues (Figure 1C). Meanwhile, the level of LINC01711 in human healthy esophageal epithelial cell line HEEC and five esophageal squamous cell carcinoma cell line EC9706, Eca109, TE-13, TE-1, and TTN were analyzed by RT-qPCR. The results showed that LINC01711 in five esophageal squamous cell carcinoma cell lines were upregulated in different degrees. The expression of LINC01711 in TE-1 cells was significantly higher than that in EC9706, TE-13, Eca109, and TTN cells (Figure 1D). Therefore, TE-1 cells were selected for follow-up experiments.

### Silencing LINC01711 inhibited the proliferation, migration, and invasion of ESCC and promoted apoptosis

**Figure 2:**
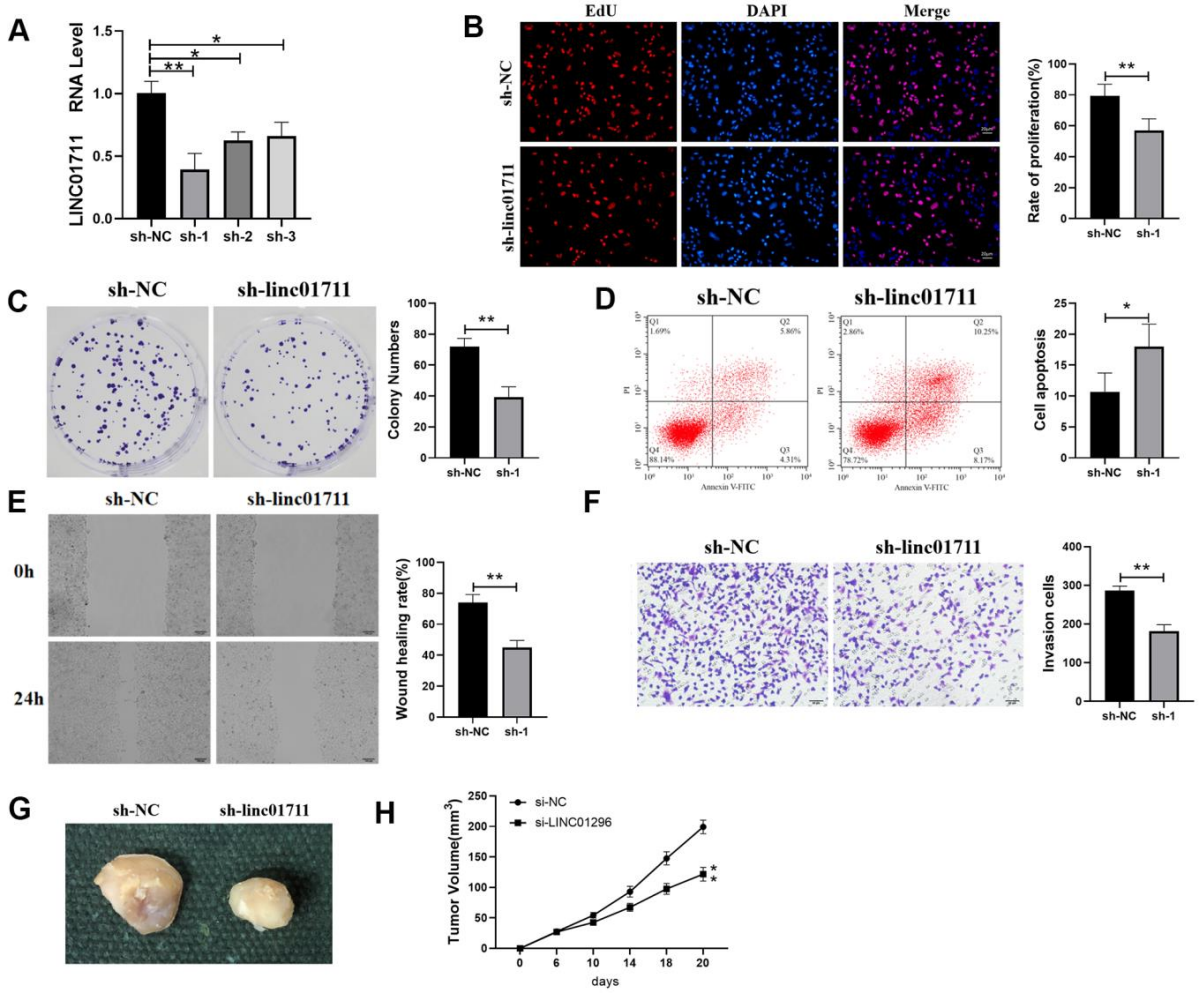
**Interference with the expression of LINC01711 inhibited the proliferation, migration and invasion of TE-1 cells and promoted apoptosis.** (**A**) RT-qPCR was used to test the interference efficiency of LINC01711 sh-RNA on TE-1 cells. n=6. (**B**) The proliferation of TE-1 cells was detected by EdU method. n=6. (**C**) Colony forming ability was determined by colony formation test. n=6. (**D**) The apoptosis of TE-1 cells was detected by flow cytometry. (**E**) The migration of TE-1 cells was detected by wound healing assay. n=6. (**F**) The invasiveness of TE-1 cells was detected by Transwell method. n=6. (**G**) Representative tumor images. (**H**) Tumor volume growth curve of each group, n=5. *p* < 0.05; ** *p* < 0.01.

To observe whether LINC01711 affects the development of ESCC, we downregulated the expression of LINC01711 using specific sh-RNA by transfection. RT-qPCR results showed that the level of LINC01711 was the lowest after transfected with sh-LINC01711-1 (Figure 2A). Therefore, in the subsequent experiment, the sh-LINC01711-1 was selected to knockdown LINC01711 and named sh-1. Then we performed the EdU assay, colony formation assay (Figure 2B, 2C), wound healing, and Transwell assay (Figure 2E, 2F) to measure the effects of LINC01711 knockdown on TE-1 cell proliferation, colony formation, migration, and invasion, respectively. We found LINC01711 knockdown resulted in the inhibition in TE-1 cell proliferation, colony formation, migration and invasion. Moreover, flow cytometry data showed that downregulation of LINC01711 promoted cell apoptosis (Figure 2D).

To test whether LINC01711 level affects tumor growth *in vivo*, we transplanted TE-1 cells in nude mice. Similarly, we found that downregulation of LINC01711 inhibited tumor growth in mice (Figure 2G, 2H). Overall, these data indicated that knockdown of LINC01711 inhibited the growth of TE-1 tumor cells *in vitro* and *in vivo*.

### LncRNA LINC01711 regulated miR-326 as a competitive endogenous RNA (CeRNA)

**Figure 3:**
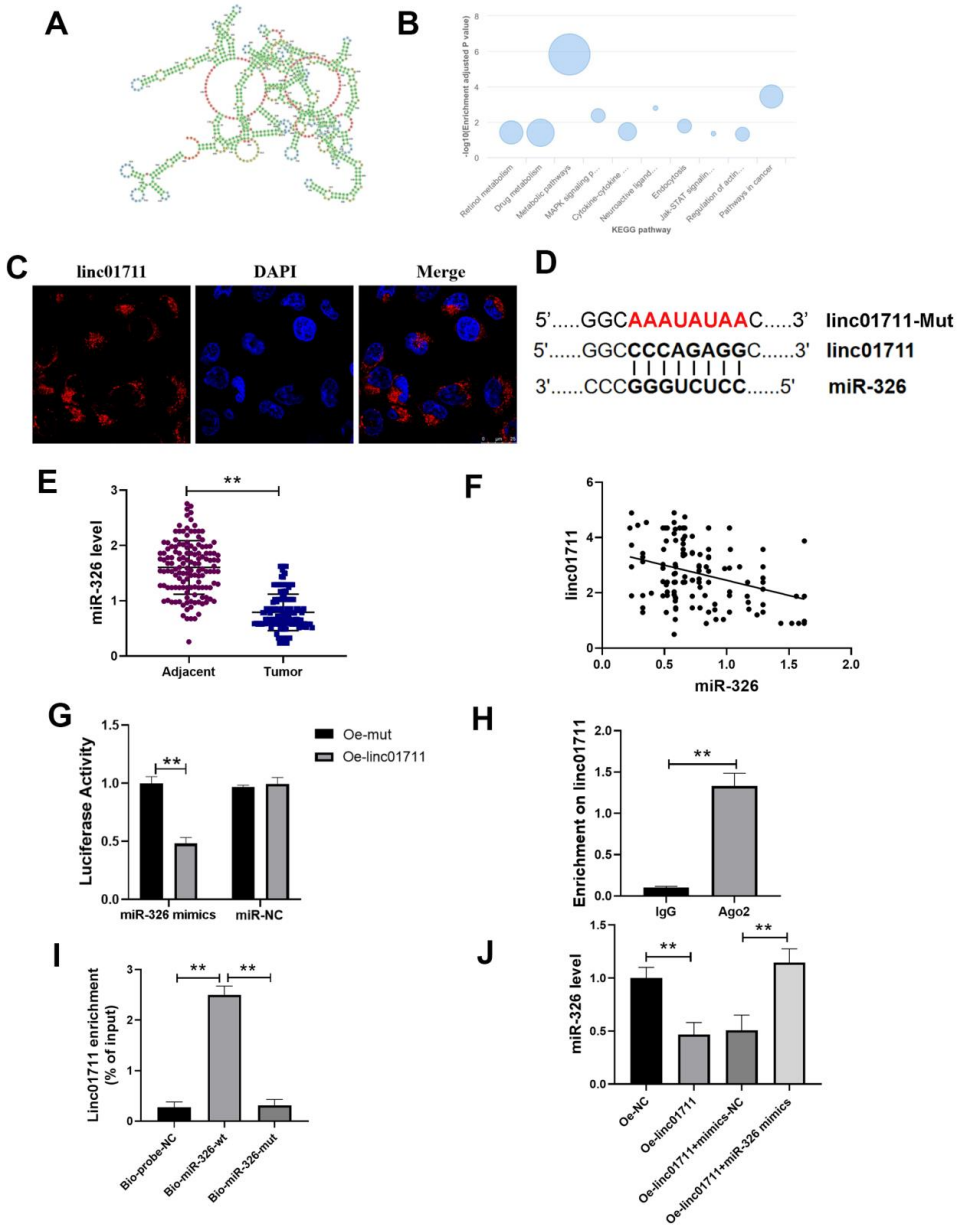
**lncRNA LINC01711 binds miR-326 as a sponge.** (**A**) Possible secondary structures of LINC01711. (**B**) Linc01711 KEGG regulatory networks predicting by LnCAR. (**C**) The LINC01711 subcellular localization was verified by FISH. (**D**) Predicted binding site of LINC01711 and miR-326. (**E**) The expression of miR-326 in ESCC and paracancerous tissues was detected by RT-qPCR. n=6. (**F**) The correlation between the expression of LINC01711 and miR-326 in ESCC was analyzed by Pearson correlation analysis. (**G**) Dual luciferase reporter assay was used to verify the binding of LINC01711 and miR-326. n=6. (**H**) The binding of LINC01711 and Ago2 was measured by RIP method. n=3. (**I**) The enrichment of LINC01711 by miR-326 was detected by RNA pull-down. n=3. (**J**) The level of miR-326 in each group was measured by RT-qPCR. n=6. ** *p* < 0.01.

In order to explore the mechanism of LINC01711 in ESCC, we predicted the secondary structure of LINC01711 (Figure 3A) and the potential pathways related to LINC01711 (Figure 3B) using the LnCAR database. Among these pathways, we found that LINC01711 was involved in the regulation of actin and pathways in cancer, suggesting that LINC01711 may regulate the migration or invasion of tumor cells. The subcellular localization of LINC01711 was detected by RNA FISH technique. The data showed that LINC01711 was mainly existed in the cytoplasm (Figure 3C). Through the prediction on lncRNASNP2 website, more than 80 potential miRNA targets were identified for LINC01711. We focused on miR-326 in our study (Figure 3D) because it has been verified as a tumor suppressor miRNA in several cancers, such as breast cancer [17] and colorectal cancer [18]. However, the function and clinical significance of miR-326 in ESCC are still at an early stage.

To verify the relation of LINC01711 and miR-326, RT-PCR was performed to analysis the expression level of miR-326 in paired ESCC tissues and normal adjacent tissues (Figure 3E). We found the expression of miR-326 was lower in ESCC tissues and was negatively correlated with the expression of LINC01711 (Figure 3F). Dual luciferase reporter assay showed that the luciferase activity of wt-miR-326 construct decreased after overexpression of LINC01711 (Oe-LINC01711), indicating that miR-326 bound to LINC01711 (Figure 3G). The AGO2-RNA immunoprecipitation experiments showed that LINC01711 had a higher specific adsorption level on Ago2 (Figure 3H), indicating the association of LINC01711 to RNA induced silencing complex. The RNA pull-down test further verified LINC01711 acted as a ceRNA and adsorbed miR-326 (Figure 3I). The free miR-326 level in the LINC01711 overexpression group was decreased, while the miR-326 mimics reversed the effect of LINC01711 overexpression on free miR-326 level (Figure 3J).

### miR-326 targets FSCN1

**Figure 4:**
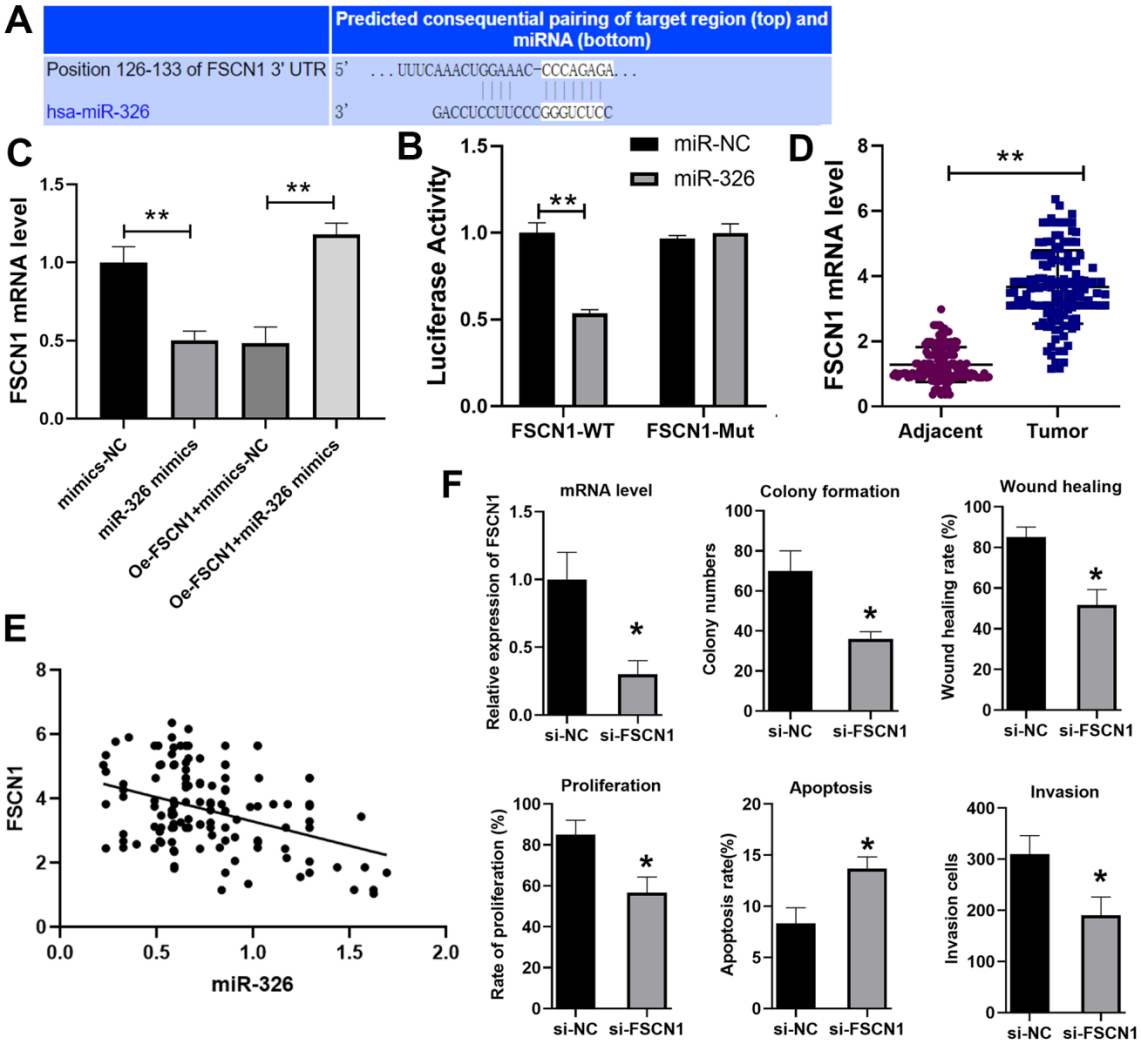
**miR-326 binds to and regulates FSCN1.** (**A**) Predicted binding site of miR-326 and FSCN1 by the Target scan website. (**B**) Luciferase reporter assay was used to verify the binding of miR-326 and FSCN1. n=6. (**C**) RT-qPCR analysis of the level of FSCN1. n=6. (**D**) The expression of FSCN1 in ESCC and paracancerous tissues was detected by RT-PCR. n=6. (**E**) The correlation of FSCN1 and miR-326 in ESCC was analyzed by Pearson correlation analysis. (**F**) Knockdown of FSCN1 expression by siRNA in TE-1 cells inhibited the colony formation, wound healing, proliferation, invasion and increased cell apoptosis. N=3-4. * *p* < 0.05, ** *p* < 0.01.

To find the target of miR-326, we used the Target scan website (http://www.targetscan.org/vert_72/) and identified fascin actin-bundling protein 1 (FSCN1) as the potential target (Figure 4A), which has been shown to promote ESCC previously [19]. Dual luciferase reporter assay verified that miR-326 bound to FSCN1 (Figure 4B). RT-qPCR showed that the expression of FSCN1 was decreased when cells were treated with miR-326 mimics, and overexpression of FSCN1 reversed the inhibition effect of miR-326 mimics (Figure 4C). We also analyzed the expression of FSCN1 in the paired ESCC tissues and normal adjacent tissues from 137 patients. The results showed that the level of FSCN1 was lower in ESCC tissues than normal tissues (Figure 4D), and was negatively correlated with the expression level of miR-326 (Figure 4E). Through siRNA knockdown, we confirmed that the pro-tumor effect of LINC01711 was mediated through modulating FSCN1 expression (Figure 4F). Reduced expression of FSCN1 in TE-1 cells by siRNA treatment resulted in inhibited colony formation, wound healing, proliferation, migration, invasion and increased apoptosis. Overall, theses data indicated that LINC01711 can inhibit miR-326 and up-regulate the level of FSCN1.

### The esophageal cancer cells transported LINC01711 through the exosome

**Figure 5:**
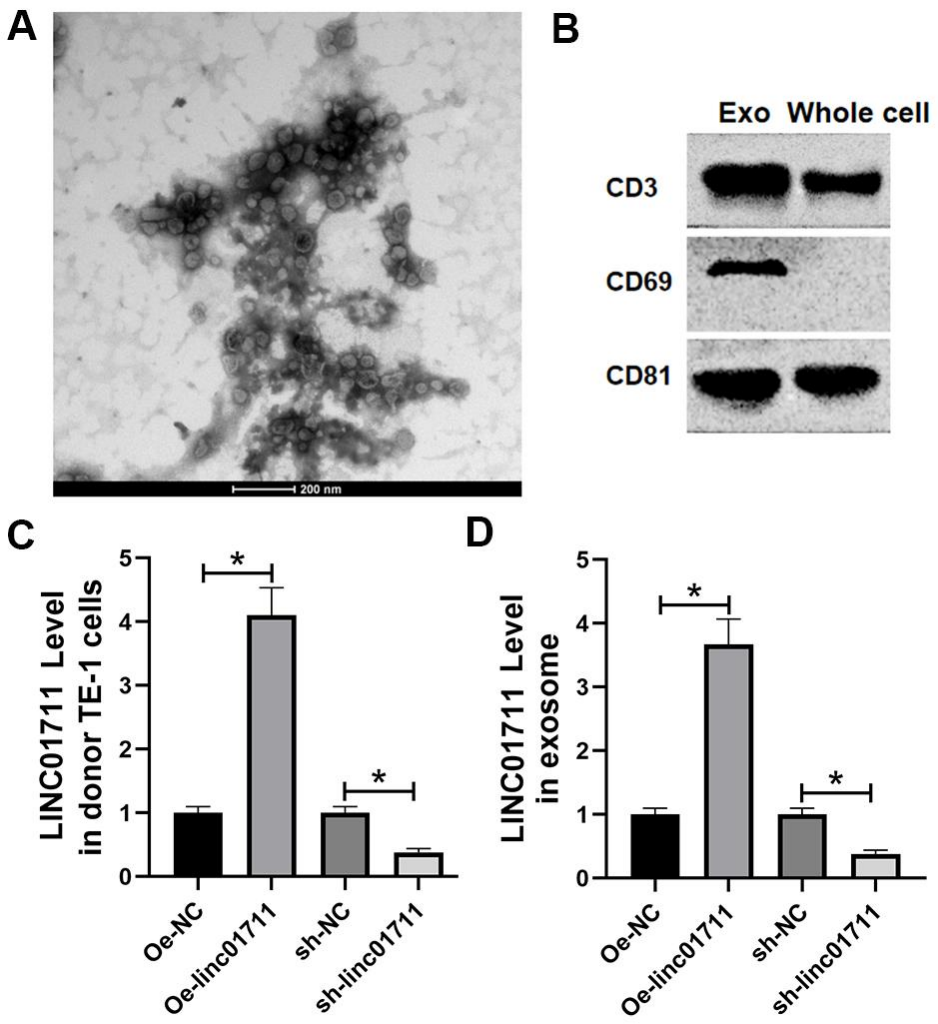
**ESCC cells transported LINC01711 to surrounding cancer cells by exosomes.** (**A**) Extracellular bodies were round or oval membranous vesicles observed by a transmission electron microscope. (**B**) The exosomes surface marker proteins CD63, CD9 and CD81 were tested by Western blot. n=3. (**C**, **D**) The level of LINC01711 in TE-1 cells and derived exosomes was measured by RT-qPCR. n=6. *p* < 0.05.

Since high concentration of lncRNAs have been reported in the exosomes, we analyzed whether the exosomes produced by TE-1 cells contain LINC01711. We first cultured TE-1 cells in exosome-depleted medium and then isolated the exosomes in the culture supernatant. Under the transmission electron microscope, we observed many round or oval microbubbles with bilayer structure, and the diameter of the vesicle was about 30 ~ 60 nm, consistent with exosome characteristics (Figure 5A). Western blot detected the exosome markers (CD63, CD9, CD81) in the isolated fractions (Figure 5B). Therefore, we confirmed that these membrane structures were exosomes.

RT-qPCR was used to test the level of LINC01711 in TE-1 cells and derived exosomes after transfection of Oe-LINC01711 plasmid and sh-LINC01711 plasmid. The results revealed that after transfection of Oe-LINC01711 plasmid, the level of LINC01711 in TE-1 cells, and derived exosomes was higher than that in NC transfected cells and isolated exosomes. In comparison, the level of LINC01711 in TE-1 cells and derived exosome and was lower in sh-LINC01711 transfected cells (Figure 5C, 5D).

### Exosome-derived LINC01711 promoted the proliferation, migration and invasion of esophageal cancer cells, and inhibited apoptosis

**Figure 6:**
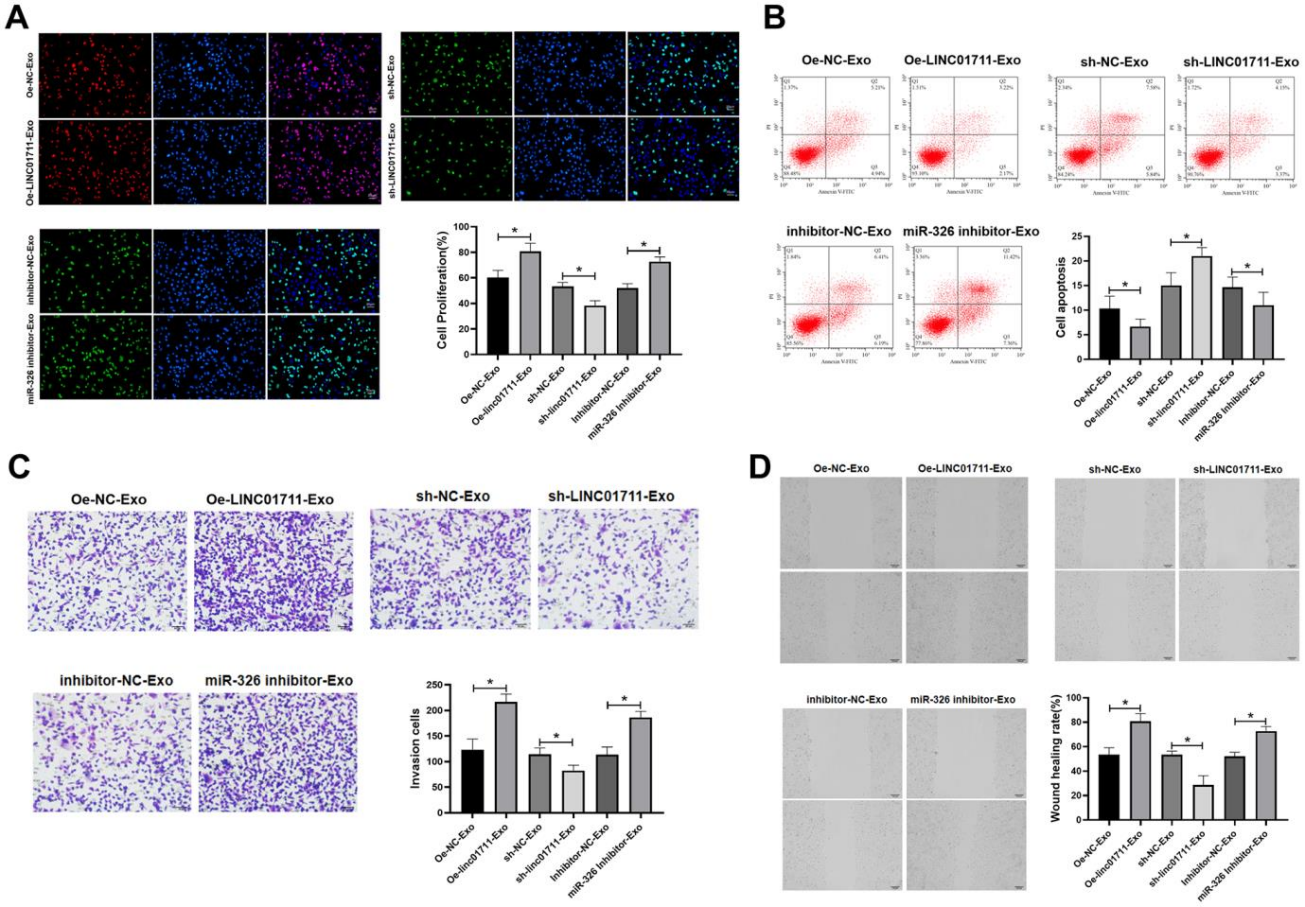
**Exosome shuttle LINC01711 down-regulated miR-326, and up-regulated FSCN1.** (**A**) Cell proliferation was detected by EdU assay. n=6. (**B**) The apoptosis of cells was detected by flow cytometry. n=6. (**C**) The invasiveness of cells was tested by the Transwell method. n=6. (**D**) Cell migration was measured by wound healing assay. n=6. * *p* < 0.05.

The effect of exosome-derived LINC01711 on receptor cells were studied by co-culture model. Oe-LINC01711 plasmid, sh-LINC01711 plasmid, and miR-326 inhibitor transfected cells were used for exosomes production. The purified exosomes were co-cultured with TE-1 recipient cells for 24 hours, and the effects were measured *in vitro* (Figure 6A–6D). The results showed that the ability of proliferation, migration and invasion of TE-1 cells treated with exosomes from LINC01711 overexpression cells (Oe-LINC01711-exo) was higher than that control treated cells, and the apoptosis was decreased in Oe-LINC01711-Exo treated cells. Similar effects were observed for exosomes from miR-326 knockdown cells. On the other hand, exosomes from LINC01711 knockdown cells showed opposite effects.

### Exosomes from LINC01711 overexpression cells promoted tumor growth in nude mice

**Figure 7:**
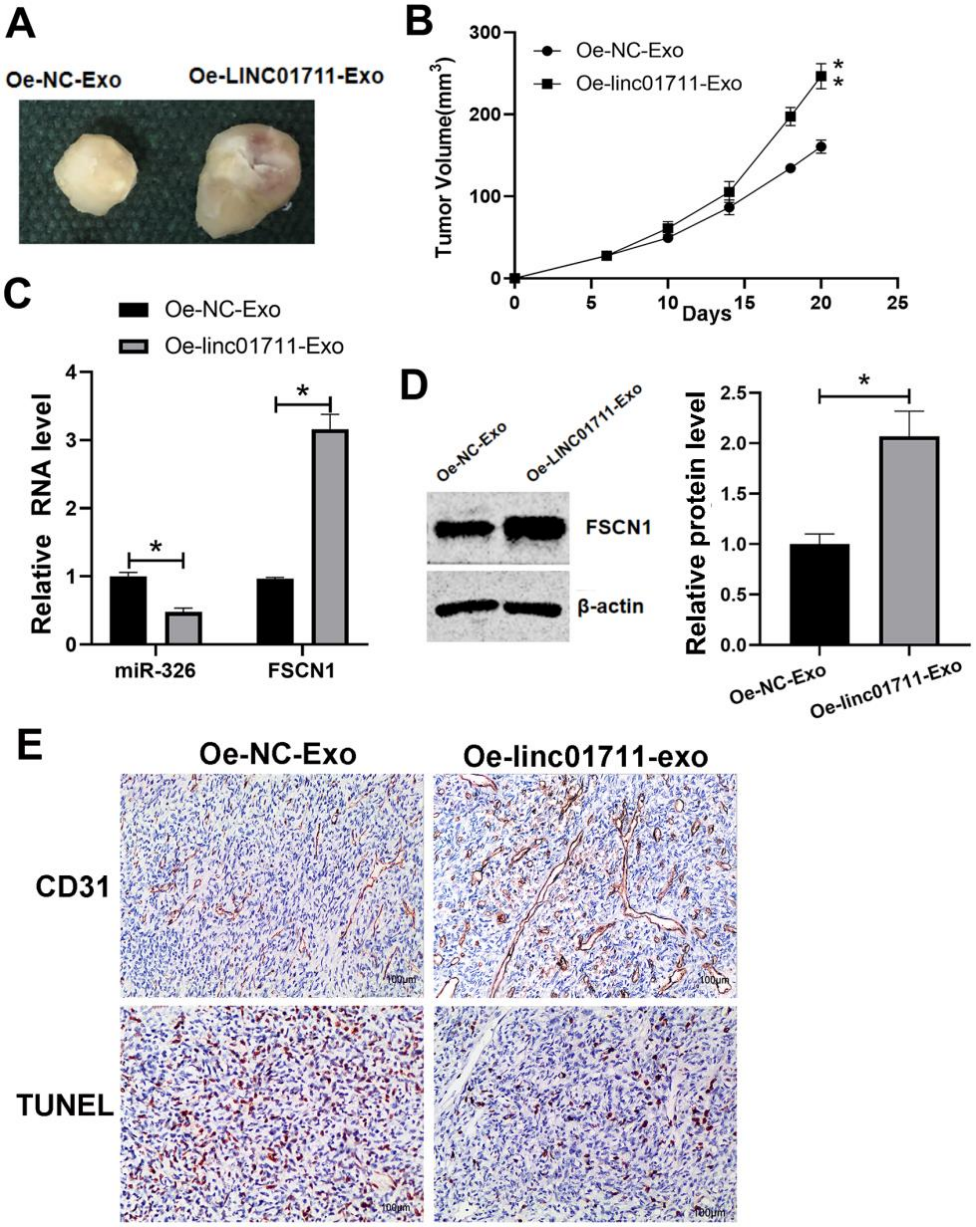
**LINC01711-Exo promoted tumor growth *in vivo*.** (**A**) Representative tumor images. (**B**) Tumor volume growth curve. n=5. (**C**) The expression of miR-326 and FSCN1 was detected by RT-qPCR. n=6. (**D**) The FSCN1 protein level was verified by Western blot. n=3. (**E**) The apoptosis and angiogenesis of tumor tissue section as detected by TUNEL assay and CD31 immunostaining, respectively. Representative images were shown. * *p* < 0.05; ** *p* < 0.01.

To test if the exosomes from LINC01711 overexpression cells could promote tumor growth *in vivo*, nude mice were injected subcutaneous with TE-1 cells and then treated with exosomes of different origins. We found that compared with Oe-NC-Exo treated mice, the tumor growth ability of Oe-LINC01711-Exo treated mice was significantly enhanced (Figure 7A, 7B). RT-qPCR showed that miR-326 in Oe-LINC01711-Exo treated mice was significantly lower than that in Oe-NC-Exo treated mice, while the FSCN1 was significantly up-regulated in Oe-LINC01711-Exo treated mice (Figure 7C). Western blot results showed that the protein level of FSCN1 in Oe-LINC01711-Exo treated mice was significantly higher than that in Oe-NC-Exo treated mice (Figure 7D). We performed staining using sections of tumor tissue and found that increased level of LINC01711 inhibited the apoptosis of tumor cells as shown by Tunel assay, and promoted angiogenesis as indicated by CD31 immunostaining (Figure 7E). To sum up, these data suggested that exosomes from LINC01711 overexpression cells promoted the tumor growth of nude mice *in vivo*.

## DISCUSSION

Effective drug targeting is critical in cancer therapy [20]; the exosome is a promising delivery system in the fight against cancer. As a carrier of intercellular cargos, such as proteins and nuclear acids, exosomes can transduce molecular signals to other cells and tissues and participate in a variety of biological events. For example, it can carry tumor genetic information, regulate tumor microenvironment and promote tumor angiogenesis [21]. LncRNA is a particular cargo of exosome and could be delivered through the exosome transfer pathway to recipient cells, causing changes in cell phenotype and leading to the transformation from normal cells to malignant phenotypes [22, 23]. The lncRNA in exosomes is stable and protected from degradation by RNases, thus promotes the cellular communication and remodeling [24–26].

In this study, we identified the novel role of LINC01711 in ESCC. LncRNA LINC01711 promoted the proliferation, migration, and invasion of esophageal cancer cells by up-regulating FSCN1 and down-regulating miR-326, and inhibiting apoptosis, thus promoted the occurrence and development of esophageal cancer. Importantly, LINC01711 existed in the exosomes originated from cancer cells, and the levels were well correlated with the intracellular concentration of donor cells. Exosomes containing different amount of LINC01711 showed proportional effects on tumor growth in an *in vivo* nude mice model.

FSCN1 was initially characterized as an actin-bundling protein, and its primary function was to bundle actin microfilaments into tight, relatively rigid, parallel bundles [27]. It plays pivotal roles in cell migration, motility, adhesion, and cellular interactions [28]. In cancer cells, aberrantly expressed FSCN1 stabilizes actin filaments in invasive foot structures and promotes the degradation of the extracellular matrix by coordinating the presentation of matrix metalloprotease, and thus promotes cancer cell growth, migration, invasion, angiogenesis and metastasis [19]. In our study, we found LINC01711 regulated the expression of FSCN1 through targeting miR-326 in TE-1 cells, which provided deeper insights into the mechanism of FSCN1 on cancer cells.

The exosomal pathway is a new way of information exchange between cells, which plays an essential role in the development of tumor cells. Although the information encoded by lncRNA was not translated into proteins, this RNA can regulate the expression of genes, cause transcripts to be degraded or inhibit protein translation, participate in the modification of epigenetic characteristics, and regulate the stability of protein complexes. The lncRNA in exosomes is stable and resistant to RNase degradation. Simultaneously, it can promote the exchange of genetic material, which has an essential impact on the biological behavior of receptor cells [24–26].

Zhang et al. [29] extracted exosomes by collecting cervicovaginal lavage samples from patients with cervical cancer and normal patients. The exosome was isolated by ultracentrifugation and the structural integrity was confirmed by a transmission electron microscope. LncRNA HOTAIR, MALAT1 and MEG3 in exosomes were quantitatively analyzed. The results revealed HOTAIR, MALAT1 and MEG3 in cervical cancer patients was higher than that in healthy patients, indicating that lncRNA HOTAIR, MALAT1 and MEG3 levels in the exudates of vaginal lavage fluid samples have the potential of being the biomarkers for early diagnosis of cervical cancer. Wang et al. [30] isolated exosomes from the serum of patients with bladder cancer by rapid purification *in vitro*. The exosome was identified by transmission electron microscope and nanoparticle tracking analysis. They found that lncRNA H19 in serum exosome was positively correlated with the H19 expression in paired bladder cancer tissues, and the level of lncRNA H19 in serum exosome in bladder cancer patients was higher than that in healthy subjects or patients with benign diseases. It was suggested that the exosomes in body fluid were a highly stable source of biomarkers of disease. Kaplan-Meier survival curve showed that the higher the level of serum exosome-derived H19, the lower the survival rate in bladder cancer patients, suggesting that the level of lncRNA H19 in exosomes is of value in predicting the prognosis of bladder cancer.

At present, most clinical studies still detect the expression of lncRNA from tumor tissues. This method is traumatic and can not achieve the minimally invasive diagnosis and recurrence monitoring of tumors. Based on the results of this study, the detection of LINC01711 in body fluid exudates can be used as a non-invasive diagnosis and monitoring index for patients with ESCC, which simplifies the steps of diagnosis and improves the efficiency. Our study supported novel methods for early diagnosis, postoperative recurrence, regular reexamination and monitoring of cancer based on the exosomes derived LncRNA biomarkers.
